# The Defeathering Effect by Scalding in Chickens Follows Their Intrinsic Dermal Histologies

**DOI:** 10.3390/ani13162584

**Published:** 2023-08-10

**Authors:** Chia-Cheng Shung, Kun-Yi Hsin, Fa-Jui Tan, Shuen-Ei Chen

**Affiliations:** 1 Department of Animal Science, National Chung Hsing University, Taichung 40227, Taiwan; xjc60@yahoo.com.tw (C.-C.S.); kunyi.hsin@nchu.edu.tw (K.-Y.H.); 2 The iEGG and Animal Biotechnology Center, National Chung Hsing University, Taichung 40227, Taiwan; 3 i-Center for Advanced Science and Technology (iCAST), National Chung Hsing University, Taichung 40227, Taiwan; 4 Innovation and Development Center of Sustainable Agriculture (IDCSA), National Chung Hsing University, Taichung 40227, Taiwan

**Keywords:** chickens, scalding, skin histology, collagen denaturation, follicle sheath

## Abstract

**Simple Summary:**

The intrinsic differences of skin histology between the feather calamus and the surrounding outer follicle sheath and neighboring cutaneous tissues reflect their resistance to thermal denaturation and account for the defeathering effect by scalding in various breeds and ages of chickens.

**Abstract:**

This study aimed to delineate the fundamental skin histology and its association with feathers in broilers and native Red-Feather (RF) chickens and further elucidate their thermal alterations in respect to the defeathering effect by scalding. Comparisons of skin thickness between fresh samples and those after dehydration and fixation, as well as their collagen contents and histological differences, suggested that RF chickens had a thicker dermal layer with more collagen deposition and compact architecture, particularly in the neck and abdominal skin, but a thinner hypodermal layer in the back, chest, and abdomen skin. Despite an adolescent age, RF chickens showed a shorter calamus depth of tail feathers but a larger calamus diameter of wing feathers. Within the feather follicle punch, a very intense follicle sheath layer with compact collagenous matrixes to fulfill the space next to the inner feather root sheath was observed in RF chickens. Under both soft and hard scalding, RF chickens showed a lower degree of denaturation on hip skins and were more resistant to structural disintegration, primarily within the epidermal and dermal layer. Accordingly, a much narrower gap space between the feather sheath and surrounding follicle sheath was observed, and the gap expansion was also resistant to thermal changes. These results suggest that the defeathering effect by scalding follows the intrinsic skin histologies in chickens of various breeds and ages, primarily depending on the interaction of the feather calamus with the surrounding follicle sheath and neighboring cutaneous tissues, reflecting their resistance to thermal denaturation, but is irrelevant to the feathers per se.

## 1. Introduction

During the commercial slaughter of poultry, defeathering is performed by dipping carcasses into hot water, namely scalding, for a controlled duration and temperature to loosen the skin and then immediately picking the feathers by rotating carcasses in an automatic feather-plucking machine. In the standard scalding procedure, the scalding tank holds a device to flip over the carcasses and normally can remove up to 80% of feathers of the carcass, thus greatly reducing the force and duration required in the feather-plucking process. An increase in submersion duration or temperature during scalding can facilitate feather removal but also affect the appearance, color, and cooking characteristics of carcass skin and meat [1,2]. Both submersion duration and temperature differentially affect carcass quality depending on the altered amplitude of the two parameters. Therefore, the defeathering effect by scalding and carcass quality should be balanced.

The scalding process for chicken defeathering in commercial slaughter can be categorized into hard and soft scalding depending on the scalding duration and temperature. Hot scalding is typically conducted at 60–66 °C for 45–90 s, and soft scalding is usually performed at lower temperatures but over a longer duration, such as 54–58 °C for 60–120 s [3]; when a temperature of 51–54 °C is used, the required submersion duration is further extended to 120–210 s [4,5]. Occasionally, scalding conducted at 54–58 °C for 60–120 s is classified as medium/sub-scalding [3,6]. An increase in duration and force during the feather-plucking process can achieve a more complete defeathering effect, but it also increases mechanical damages on carcass appearances, and thus a high scalding temperature and minimal feather-plucking time is recommended [7,8,9].

A considerable part of chickens is sold as whole carcasses in the market, particularly for ceremony in Asian countries. Carcass appearance, particularly skin color, attracts the first sight of consumers and determines their choice of purchases. Red Feather (RF) chickens are a strain of commercial native chickens in Taiwan, accounting for 20% of chicken meat production annually. The RF chickens are marketed around 2.6–2.9 kg of bodyweight (BW) at an age of 12 weeks as compared to the rapid-growing commercial broilers (2.0 kg BW within 5 weeks) [10]. Due to no official protocol for commercial slaughter of RF chickens, we conducted a series of studies and recommended hard scalding at 60 °C for 60 s for the slaughter procedure of RF chickens for a better defeathering effect without unfavorable changes of carcass quality [11].

In addition to the recommended scalding method using juvenile broilers and adolescent and adult RF chickens with a similar body weight but old layer chickens at a much lower BW, we concluded that the defeathering effect by scalding depends on the age to reach a certain threshold in the development of skin architecture and feather richness and is irrelevant to body weight, while the alteration of carcass quality, particularly skin color, is primarily determined by age, body weight/breed, and their interaction [11]. After scalding and the feather-plucking process, most of the feathers remaining in the carcass were found on the hips, particularly wings and tail in birds with undesirable defeather scores [11].

Feathers develop and grow from the follicles in the skin. When feathers reach their maximum size, they remain attached to the follicle until replaced during molting [12]. Each feather is fixed through its extremity, the calamus, in a small cavity invaginated into the skin (follicle punch), and the invagination even implants vertically down to the hypo-dermal layer [13,14,15]. Within the follicle, the dermal papilla containing capillary networks protrudes into the pulp at the bottom of the feather calamus, while the feather sheath of the calamus (inner feather root sheath, a highly keratinized structure on the surface of feather calamus) is bound by the surrounding follicle sheath (outer feather root sheath, a less keratinized structure extending from the follicle epidermis) [14]. Interactions between the camalus and surrounding follicle sheath, as well as with neighboring cutaneous collagenous matrixes, render the feather anchored tightly on the skin.

Although scalding methods have been used for a long time in commercial poultry slaughter, very few studies have been conducted to elucidate the fundamental architecture of skin histology of chickens and its interactions with associated feathers in respect to various strains and ages, much less their alterations by temperature. Accordingly, the present study aimed to delineate the intrinsic histology of different parts of skins in RF chickens using broilers at comparable BW as a reference and further examine their thermal alterations under soft and hard scalding processes. Results of this study provide the fundamental histology of skins and their alterations with associated feathers to elucidate the defeathering effects by scalding methods.

## 2. Materials and Methods

### 2.1. Animals

To exclude the sex effect, only female chickens were used in the study. A flock of adolescent (12-weeks-old) female RF country chickens at market weight around 2.7 kg were purchased from a local farm and transported to the Animal Experiment Farm of National Chung Hsing University 1 day before slaughter. A flock of broilers (Ross 308) were raised to the age of 7 weeks to achieve BW comparable to the RF chickens (Table 1). The chickens were fasted for 12 h before the experiment and shipped to the electric slaughterhouse 3 h before slaughter. The slaughter procedure followed the standard protocol (i.e., electric water-bath stunning, bloodletting, and scalding). Birds were sampled immediately after bloodletting or after scalding without further processing. The slaughter process was conducted in the poultry slaughterhouse located beside the Livestock Experimental Farm.

### 2.2. Slaughter Process and Sample Collection

Each strain comprised 30 female chickens, of which 10 birds were used for skin sampling immediately after bloodletting, while the other 20 birds were subjected to soft or hard scalding process (*n* = 10 for each) by submerging in water bath at 57 °C for 120 s or at 60 °C for 60 s, respectively. The slaughter process was as follows: the chickens were first weighed, after which their feet were hung upside down in a shackle of the automatic transport chain. Subsequently, the chickens were stunned by a direct current (voltage, 50 V; current, 20 mA) for 12 s in an electric water bath, and thereafter the carotid artery and jugular vein were cut open for bloodletting for 120 s [11]. The scalding tank holds an automatic temperature and time-setting function, a steam circulation heating system, and a 360° continuous flipping and soaking mechanism (capacity of 7 chickens per scalding). Skin samples in 1.5 × 1.5 cm^2^ at indicated positions on the head, neck, back, chest, abdomen, and hips as well as cutaneous tissues in the wings and tail were collected as described in the following methodologies.

### 2.3. Histology Analysis

Collected skin samples from each part were fixed and dehydrated in 10% buffered neutral formalin for 7 days. After fixation, tissues were moderately cut, trimmed, and then placed into embedding cassettes. The cassettes were then filled with paraffin for embedding. The embedded tissues were sent to the Histology Service of the National Chung Hsing University, Taiwan, for hematoxylin and eosin (H&E) and Masson’s trichrome staining [16,17,18]. A microscopy and photomicrography imaging system (SGI mage V 2.1) manufactured by SAGE Vision was employed for observations and measurements.

### 2.4. Skin Thickness and Collagen Quantification

**Figure 1:**
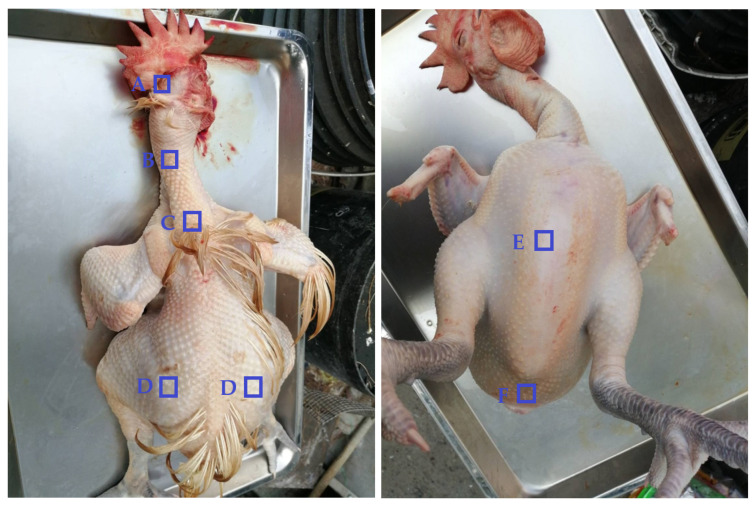
The positions of skin sampling. Immediately after bloodletting or after scalding, the skin of the head (right below the comb), neck (the middle point between the sampling position of head and back skin), back (the middle point between right and left wing), chest (the most protruding site of the sternum bone), abdomen (at the middle point), and hips (at the joint between femur and ischium) of broilers and RF chickens were collected as shown in blue squares. A. head. B. neck. C. back. D. hip. E. chest. F. abdomen.

Before scalding, the skin (in 1.5 × 1.5 cm^2^) of the head (scalp; right below the comb), neck (the middle point between the sampling position of head and back skin), back (the middle point between right and left wing), chest (the most protruding site of the sternum bone), and abdomen (the middle point) were collected at the positions shown in Figure 1. Collected skin samples were soaked in 10% formalin for fixation and then for H&E and Masson’s trichrome staining [17,18]. The thickness measurements under microscopic observations were made at 3 points per image at the middle site and the sites next to the right and left edge peduncular to the stromal tissues. Three images per bird and 10 birds per strain were used for skin thickness measurement under microscopic observations. An additional piece (1.5 × 1.5 cm^2^) of each part of skin was collected, spread out in a plastic folder by stapling, and then frozen at −20 °C for 24 h. In the next day, the thickness of the longitudinal section of the frozen skin sample was immediately measured using a vernier caliper. Each sample was measured at 9 different points in every 0.25 cm^2^ area throughout the skin pieces. The results were compared to those after fixation under microscopic measurements. Masson’s trichrome staining was performed for collagen visualization, and the chromogenic intensity was used for quantification using Image-J software (1.44, NIH, Bethesda, MD, USA). Three images per bird and ten birds per strain were used for skin collagen quantification.

### 2.5. Diameter and Depth of the Calamus of Wing and Tail Feathers

After bloodletting and prior to scalding, the 1st and 5th secondary feather of both right and left wing and those of the main tail feathers (not the sickle feathers) were used for diameter and depth measurement of the feather calamus. The feather shaft was marked at the site above the surface of surrounding skins and then cut off. The remaining feather shaft regarded as the calamus was then retrieved using pilers. Subsequently, the diameter of the feather calamus was measured at the cross section of the feather calamus, and the feather calamus length was measured from the mark site peduncular to the bottom of the calamus.

### 2.6. The Gap Distance between the Feather Calamus Shaft and Surrounding Cutaneous Tissues

Immediately after bloodletting (10 birds for each strain), or after soft or hard scalding before feather plucking (10 birds for each strain), the 3rd and 7th feather of both right and left secondary wing feathers and those of the main tail feathers (not the sickle feathers) were cut off at the site above the skin surface, and the surrounding cutaneous tissues containing the feather follicle and remaining feather calamus were collected for H&E staining [16,17]. The gap distance between the feather sheath surface and surrounding follicle sheath was measured under microscopic observations. Six images of each feather from the top (right beneath the skin surface), middle, and bottom site (at the U-turning point along the shaft surface of the calamus) around the feather punch (right and left side) were used for the gap distance measurement. Each image was measured at 3 points, from the middle site to the site next to the right and left edge.

### 2.7. Denaturation of Dermal Structures of the Hip Skins

Collected hip skin samples (at the joint between femur and ischium as shown in Figure 1) immediately after bloodletting or after soft or hard scalding were fixed in 10% formalin for H&E staining. The distance of color changes from the outer epidermal (dark purple) to the inner dermal layers (bright pink purple) was measured for the degree of denaturation by scalding, and the integrity of epidermal and dermal structures were imaged under microscopy [18].

### 2.8. Statistics

Data were first analyzed by Bartlett’s and Shapiro–Wilk test for homogeneity of variance and normality and then by one-way analysis of variance (ANOVA) using breed or scalding method as the variable. A two-way ANOVA using scalding condition (hard vs. soft) × breed (broilers vs. RF chickens) as variables was performed to analyze the main effects and their interaction on skin denaturation. Data were presented as mean, and comparison results by student’s t-test or Tukey’s range test with *p* < 0.05 level regarded as a significant difference. The statistics were performed by Statistica 7.0 (Statsoft, Tulsa, OK, USA).

## 3. Results

### 3.1. Skin Thickness and Collagen Contents

Broiler and RF chickens at an age of 7 and 12 weeks, respectively, with similar BW were used in the study (Table 1). Different parts of skin throughout the body including head, neck, back, hips, chest, and abdomen were sampled for skin histology immediately after bloodletting or after scalding process (Figure 1).

**Figure 2:**
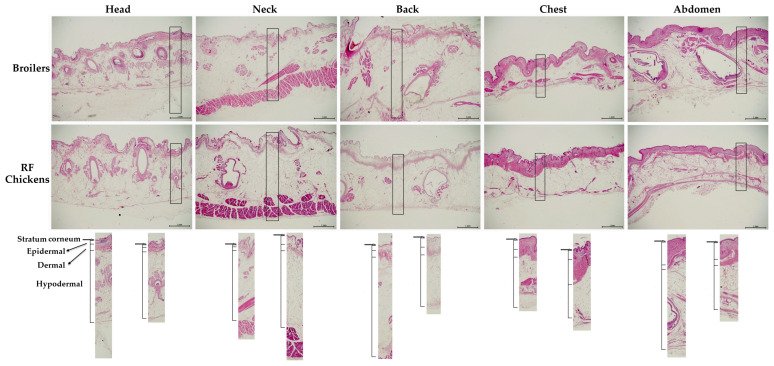
The histology and thickness of different parts of skin in broilers and RF chickens. Before scalding, the skin of different parts of the body were collected as shown in Figure 1. Collected skin samples were soaked in formalin for fixation and then for H&E staining. The skin layers were ordered as stratum corneum, epidermal, dermal, and hypodermal layer from the exterior to interior. Scale bars indicate 1 mm.

Among the fresh skin samples (F), despite an older age, RF chickens exhibited a significant lower skin thickness on the back but higher on the abdomen than that of broilers (*p* < 0.05, Table 2). Under microscopic observations with the skin samples after dehydration and fixation (D), both back and abdomen skins showed a lower thickness in RF chickens, while only the abdomen skin showed a lower D/F ratio (*p* < 0.05, Table 2). Among the various layers of fixed samples, RF chickens showed a thicker stratum corneum layer on the neck and back and thicker dermal layer on the head (scalp), neck, back, and chest skin but a thinner hypodermal layer in the back, chest, and abdomen skin, while no differences in the epidermal thickness throughout the body were observed between the two breeds of chickens (*p* < 0.05, Table 3, Figure 2).

**Figure 3:**
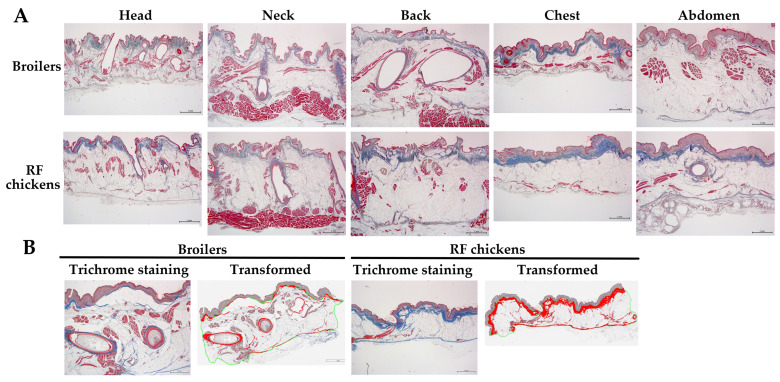
Distribution of collagen of different parts of skin in broilers and RF chickens. Immediately after bloodletting, collected skin samples before scalding process were fixed in formalin and then used for Masson’s trichrome staining (panel (**A**)). The chromogenic stainings (blue) were transformed into areas and used for collagen quantification under Image-J software (panel (**B**), represented by abdominal skins). Scale bars indicate 1 mm.

Under Masson’s trichrome staining, most skin collagens were distributed throughout the dermal layer and to a less degree on the bottom edge of the hypodermal layer (Figure 2). RF chickens exhibited more rugged surfaces with bumpy ridges and ravines on the stratum corneum layer, particularly on the head (scalp), neck, and chest skin (Figure 2 and Figure 3). Furthermore, more collagens scattered throughout the hypodermal dermal layer were observed in RF chickens (Figure 3). Quantification with the histological staining suggested that RF chickens had a higher abundance of collagen in the neck and abdominal skin (*p* < 0.05, Table 4). In combination with the results from Table 2 and Table 3, RF chickens apparently have more collagen deposition and thus hold more water in the interstitial space within the dermal layer of neck and abdominal skins, while the interstitial networks of collagenous matrixes are structured in a more compact manner [16,17], probably due to their older age.

### 3.2. Feather Calamus Length and Diameter

**Figure 4:**
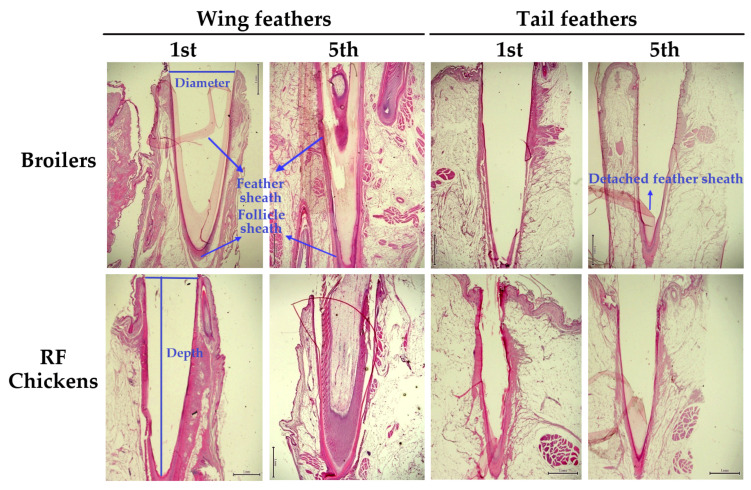
The morphology of feather follicle and calamus depth and diameter in broilers and RF chickens. After bloodletting and prior to scalding, the 1st and 5th feather of both right and left secondary wing feathers and those of the main tail feathers were used for feather calamus depth and diameter measurement. The feathers were cut off above the surrounding skins and the remaining feather calamus was retrieved for diameter and depth (length) measurement. The feather calamus containing associated cutaneous tissues were collected for K & E staining. Scale bars indicate 1 mm.

Most of the remaining feathers after scalding process were observed in the wing and tail [11]. We then examined whether the feather per se, in respect to its size, namely calamus depth and diameter, is associated with the defeathering effect by scalding. Surprisingly, despite an adolescent age compared to juvenile broilers, RF chickens showed a shorter calamus depth of the first main tail feather (MTF) but a larger calamus diameter of the fifth secondary wing feather (SWF) (*p* < 0.05, Table 5, Figure 4), while the other feathers were not different from those of broilers. Histological examination showed that within the feather follicle punch, a very intense follicle sheath layer full-filled with collagenous matrixes is compacted in the gap space next to the inner feather sheath (Figure 4). The follicle sheath (outer feather sheath) layer initiates from the epidermis down to the bottom of the feather calamus. These results suggest that the retention of wing and tail feathers after scalding largely depends on the skin histology and interactions of the feather calamus with the surrounding outer feather sheath layer, as well as with neighboring cutaneous tissues, but is irrelevant to the feather per se, including size and length as influenced by age.

### 3.3. Skin Denaturation and Histological Alterations in the Feather Follicle and Surrounding Cutaneous Tissues by Scalding

**Figure 5:**
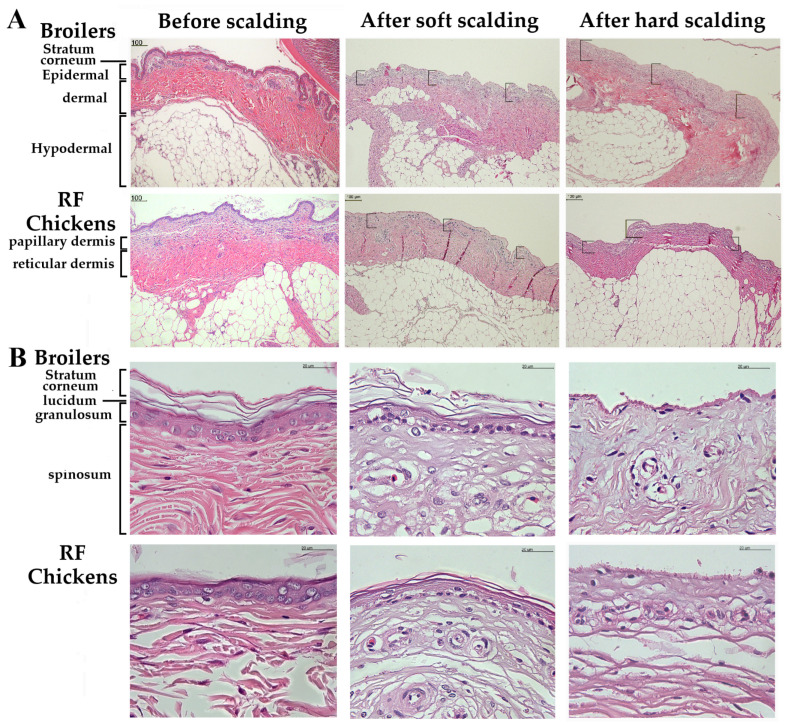
Effects of scalding on the denaturation of hip skins in broilers and RF chickens. Collected hip skin samples immediately after bloodletting or after soft or hard scalding were fixed in formalin for H & E staining. The denaturation by scalding was observed by color changes from the outer epidermal layer (dark purple) to the inner layer (bright pink purple, panel (**A**)). The integrity of epidermal and dermal structures was imaged under a microscopy (panel (**B**)). Scale bars indicate 100 μm in panel A and 20 μm in panel (**B**).

The alterations of skin color in chicken carcasses have been shown mainly relying on the age, breed, and their interaction [11]. In H & E staining, the color change from the outer epidermal (dark purple) to the inner dermal layer (bright pink purple) can reflect the degree of skin denaturation by temperature [18]. We then examined skin denaturation by measuring the range of color change along the skin layers before and after scalding. Scalding process altered the staining color as observed from the outer epidermal layer gradually into to the dermal layers (Figure 5). The two-way ANOVA suggested that scalding method and breed significantly affected hip skin denaturation, but without an interaction by the two factors (*p* < 0.05, Table 6), in which RF chickens exhibited a lower degree of denaturation under both soft (57 °C for 120 s) and hard scalding (60 °C for 60 s), while hard scalding exerted a more pronounced effect in both breeds of chickens (*p* < 0.05, Table 6, Figure 5).

In the histological alterations, scalding process, particularly hard scalding in broilers, detached the stratum corneum layer and remarkably disintegrated the array of stratum lucidum, granulosum, and spinosum in the epidermal layer, and papillary and reticular layers in the dermis, and even destroyed their architectures (Figure 5). In contrast to RF chickens, the epidermal granulosum layer in broilers was completely abolished. Since only the abdomen skin showed a very pronounced collagen abundance in RF chickens (Table 4), the resistance of skin to thermal denaturation thus is attributed to the architectural strength of the collagenous networks.

**Figure 6:**
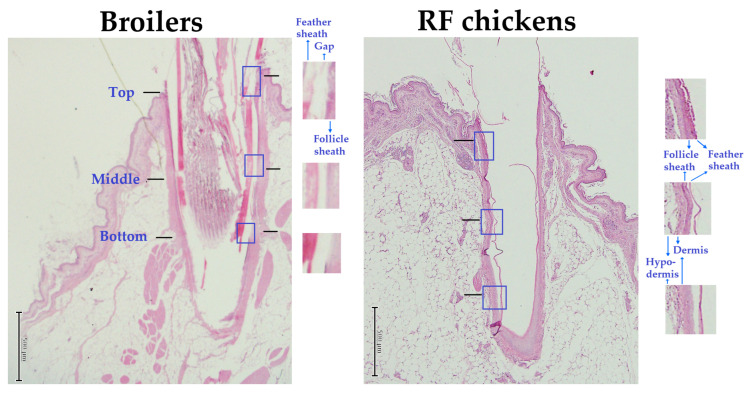
Histological alterations by scalding on the feather follicles of wing and tail feathers in broilers and RF chickens. Immediately after bloodletting or after soft or hard scalding, the 3rd and 7th feather of both right and left secondary wing feathers and those of the main tail feathers were cut off. The feather follicle containing the feather and associated cutaneous tissues were collected for H&E staining. The gap distances between the feather sheath surface and follicle sheath were measured under microscopic observations. The 7th main tail feathers from broilers and RF chickens after hard scalding were used for representative images. Scale bars indicate 500 μm.

We then examined the histology of the feather follicles and the interaction of the associated calamus shaft with surrounding cutaneous tissues. Regardless of scalding or not, RF chickens had a much smaller gap between the feather sheath and surrounding follicle sheath (*p* < 0.05, Table 7, Figure 6). Scalding process significantly expanded the gap distance, particularly in broilers after hard scalding (*p* < 0.05, Table 7). Hard scalding even detached the follicle sheath from surrounding dermis in some broilers (Figure 6). In contrast to broilers, therefore, RF chickens exhibited resistance in skin degeneration and gap expansion between feather and follicle sheath by scalding. These results indicate a higher interaction of collagenous matrixes within the feather follicle and with the neighboring connective tissues, and thus a higher viscoelasticity to retain the feathers against mechanical force for defeathering.

## 4. Discussion

Using different breeds of chickens at various BW and ages, we concluded that the defeathering effect by scalding is governed by the age to reach a certain threshold in the development of dermal architecture regardless of BW, while the alteration of carcass color primarily depends on age, body weight/breed, and their interaction [11]. Here we further showed the fundamental skin histology in chickens and its association with feathers in respect to their thermal alterations to elucidate the defeathering effect by scalding process. The differential defeathering effects by scalding in chickens of various breeds and ages were attributed to their intrinsic differences in skin histology and the interaction of feather calamus with the surrounding feather sheath layer and cutaneous tissues, which reflect the resistance to thermal denaturation. The collagenous matrixes in the skin and within the feather follicle in RF chickens are resistant to thermal degeneration and thus more deniable to defeathering effects by scalding. Accordingly, it is interesting to determine the threshold age that birds develop a more resistant architecture in skin and feather follicle histology against thermal disintegration.

In H & E staining for histological studies, hematoxylin preferentially binds to basophilic structures such as chromatins and ribosomes containing nucleic acids and thus presents in blue-purple color, while eosin counterstains the basic elements including cytoplasm, muscle, elastin, and collagen in various degrees from pink, orange, to red color [17]. Under pathological conditions such as degeneration or due to senescence, the acidophilic cytoplasm is stained with a saturated hue of red color, while denatured collagens show darker red [17,18]. Based on the methodology, scalding process changed H&E staining color from the epidermal layer with dark-purple color gradually into the bright pink- purple dermal layers, suggesting that a high temperature damages the structures of collagenous matrixes and alters inter- and intra-molecular interactions such as hydrogen bonding, Van der Waals forces, hydrophobicity, and covalent disulfide bonds, leading to increased free thiols and pH changes and thereby staining with darker red color.

Collagen fibrils are composed by collagen I and III in the skin, and their mechanical properties largely depend on covalent crosslinks including disulfide bonds, N(g-glutamyl)lysine isopeptide, oxidation of lysine residues into allysine for crosslinking, and advanced glycation end products [19]. Moreover, the thermal stability of collagens is highly associated with their hydroxyproline contents [20]. Since type I collagen accounts for 80–90% of collagens in the skin and a domain within its triple helix form is devoid of hydroxyproline, which is required for hydrogen bonding to stabilize the triple molecule, skin thus is susceptible to thermal damage [19,20]. Thermally induced alterations occur in the collagen molecule itself in a triple helix form, as well as in the semicrystalline fibril, which is assembled by collagens side-by-side in a staggered manner along the long axis [19]. Under thermal denaturation, the intramolecular crosslinks are broken and the collagen transits from a highly organized crystalline structure to a random gel-like state [21,22]. Collagen shrinkage or hydrothermal strain/stress is a mechanical alteration by the cumulative effect of the relaxed triple helix due to the destruction of the heat-labile intramolecular crosslinks and the residual tension of the heat-stable intermolecular crosslinks [22]. The residual tension is associated with viscoelastic behaviors in the skin [21,22]. Thermal effects on collagen shrinkage can be reversible or irreversible depending on the content of collagens and their staggered manner, temperature and exposure time and the mechanical stress applied to the skin [22]. In pig ear skins, the elastic force decreased gradually as temperature increased but abruptly dropped after 60 °C, at which point thermal injury occurred within 10 s [21,23]. In accordance with the results, the relaxation response of skin tissue under thermal stress, namely viscoelasticity, is independent of temperature within a lower range (below 30 °C), but at higher temperatures, particularly after 60 °C, the skin relaxes rapidly and reaches an equilibrium state much sooner than those around body temperatures [21]. These results manifest a critical temperature point, 60 °C, around which scalding process can remarkably alter the viscoelasticity of carcass skin. The skin tensile strength has been shown to affect skin tearing in a sex-, diet-, skin thickness-, and age-dependent manner [24,25].

Fourier-transform infrared attenuated reflectance mode (FTIR-ATR) spectroscopy analysis with chicken breast meat under various scalding conditions suggested that β-sheet and β-turn structure decline gradually from 54 °C for 210 s to 57 °C for 120 s and then increase at 58 °C for 90 s, whereas α-helix configuration increases first and then decreases [19]. Under FTIR spectra analysis, the major thermal alterations of turkey collagen extracts were shown at the C=O stretch and N–H bend coupled with the C–N stretch and CH_2_ bend [26]. These alterations increased the stiffness but were adversely associated with viscoelasticity in the skin [27]. Under differential scanning calorimetry (DSC) analysis, more crosslinks in the integral collagens indicate higher denaturation temperatures and thus higher enthalpy values [28]. The DSC analysis showed that turkey collagen extracts started to be gelatinized (denatured) at 25 °C and crystallized around 63 °C due to a loss of the bonded water [26]. In chickens, the denaturation temperature (T_d_) of purified collagens started at 41 °C [29]. In a study of 2 × 2 factorial design with breed and BW as variables, the collagen dispersions prepared from the skins of heavier birds had higher integrity than those from the lighter birds, and the enthalpy value also consistently increased with age of both chicken strains [28]. Previously, we reported that the breast skin tensile force was higher in adolescent RF chickens than that of juvenile broilers under soft and hard scalding conditions, while RF chickens were resistant to the decrease in breast skin tensile force by hard scalding [11]. Since the collagen content and thickness of the chest skin were not different between broiler and RF chickens, the resistance to thermal denaturation in RF chickens thus is attributed to the collagen per se, including organized structures and their interactions with interstitial matrixes.

The epidermis is composed by keratinized stratified squamous epithelium in a stack from deep to superficial, including the stratum basale (also called the stratum germinativum), spinosum, granulosum, and corneum layer [30]. The cells in the stratum basale are bond on the underlying dermis via intertwining collagen fibers into the basal lamina, where finger-like projections, known as the dermal papillae, on the superficial edges of the dermis outgrow into the basal lamina. Dermal papillae increase the strength of the connection between the epidermis and dermis. In birds, the dermal papilla containing capillary networks protrudes as a pulp into the bottom of the feather calamus. Near the bottom of the calamus in the feather follicle, the less keratinized follicle sheath layer stretches upwards to cover the root shaft forming the inner feather sheath layer, a highly keratinized structure on the surface of the calamus shaft [12,13,14,15]. Feathers are an epidermal appendage of birds, composed by keratins in a highly arranged structure [31]. Keratins tend to aggregate into basic macromolecules due to enrichment with cysteine residues to form covalent disulfide bonds that crosslink with the adjacent matrix molecules [32]. Keratins are categorized as soft and hard keratins, depending on the amount of sulfur and the ability to associate with adjacent matrixes [33]. Soft keratins, enriched with α-keratin proteins, are organized in weakly consolidated spiral coils mostly through α-helix structures, and typically form the stratum corneum of the epidermis and hairs. Hard keratins present in the feathers, beaks, and claws of birds and have a high proportion of β-keratins, whose pleated sheets form parallel or antiparallel β-strands to tightly hold the lateral polypeptide chains together through intermolecular hydrogen bonds. Within the feather follicle, α-keratin sheath cells transit into β-keratin keratinocytes along the shaft axis and around the circumference of the calamus [34]. The replacement by dense and hard β-keratins, namely cornification, in follicle sheath cells eventually forms the calamus. The transition depends on the stage of feather growth. The degree of cornification increases upwards along the feather shaft and with the horizontal axis into the bundles of the calamus shaft [34].

During the scalding process, carcass skins are loosened in the water bath at an optimal temperature and feathers are picked off at a fixed mechanical force and duration. The effectiveness of defeathering, therefore, relies on the alteration of skin histology, namely its residual viscoelastic property. RF chickens have a very intense follicle sheath layer with compact collagenous matrixes to fulfill the space next to the inner feather calamus sheath. The skin of RF chickens is more resistant to thermal degeneration in structural disintegration, and thus exhibits less collagen shrinkage and gap expansion between the feather and follicle sheath and thereby retains a higher viscoelasticity against mechanical force. As a result, the feathers tenaciously stick to the follicle sheath during the scalding process, leading to a poor defeathering effect. In contrast to the significant alteration of skin viscoelasticity at 60 °C [21], moistured feathers at 15.2 wt % showed pronounced degeneration at higher temperatures up to 69.4 °C [35]. Accordingly, in accounting for the thermal expansion of the gap between follicle and feather sheath, the alteration of collagenous matrixes within the feather follicle including those of the follicle sheath enriched with soft keratins and the neighboring dermis apparently have more contributions than the feathers per se, a rigid structure with highly organized hard-keratin architecture.

## 5. Conclusions

In contrast to broilers, the skin and collagenous matrixes within the feather follicle in RF chickens are more resistant to thermal degeneration, and thus the feathers tend to stick on the follicle sheath, leading to a poor defeathering effect by scalding. The results may be further validated in other species such as turkeys and water fowls.

## Figures and Tables

**Table 1:** Age and body weight of chickens.

Breed	Age (Weeks)	Body Weight (g/Bird)
Broilers	7	2711 ± 110
RF	12	2658 ± 91

Each breed comprised 30 female chickens, in which 10 birds were used for skin sampling immediately after bloodletting, while the other 20 birds were subjected to soft or hard scalding process (*n* = 10 for each). Results are expressed as means ± SD (*n* = 30). RF, Red-Feather country chickens.

**Table 2:** Skin thickness in different parts of the body in broilers and RF chickens.

Unit (mm)	Head	Neck	Back	Chest	Abdomen
Fresh (F)					
Broilers	2.53 ± 0.17	3.30 ± 0.27	3.70 ± 0.23	1.48 ± 0.13	3.14 ± 0.38
RF	2.59 ± 0.36	3.44 ± 0.44	2.65 ± 0.27 *	1.45 ± 0.14	3.84 ± 0.73 *
Fixation (D)					
Broilers	2.04 ± 0.17	2.36 ± 0.15	3.21 ± 0.26	1.35 ± 0.07	2.39 ± 0.33
RF	2.24 ± 0.22	2.63 ± 0.47	2.34 ± 0.18 *	1.31 ± 0.10	1.85 ± 0.18 *
D/F ratio					
Broilers	0.81 ± 0.06	0.72 ± 0.05	0.87 ± 0.06	0.91 ± 0.03	0.76 ± 0.10
RF	0.86 ± 0.05	0.76 ± 0.12	0.88 ± 0.10	0.90 ± 0.06	0.48 ± 0.06 *

After bloodletting, freshly collected skins (F) were frozen under −80 °C overnight and in the next day were used for thickness measurement using a vernier caliper. Samples after dehydration and fixation (D) were used for skin thickness measurement under microscopic observations. Results are expressed as means ± SD (*n* = 10). * significant difference vs. broilers within the same skin part and sample processing (*p* < 0.05). RF, Red-Feather country chickens.

**Table 3:** The thickness of skin layers in different parts of the body in broilers and RF chickens.

Unit (μm)	Head	Neck	Back	Chest	Abdomen
Stratum corneum					
Broilers	27.7 ± 3.8	25.5 ± 1.3	22.7 ± 2.3	27.4 ± 0.8	28.8 ± 0.8
RF	28.3 ± 1.4	30.4 ± 1.7 *	31.5 ± 1.3 *	29.3 ± 0.6	29.1 ± 2.2
Epidermal					
Broilers	114.5 ± 20	102.6 ± 9.0	120.1 ± 9.0	143.2 ± 6.5	202.6 ± 38
RF	131.9 ± 28	122.8 ± 12	119.3 ± 20	153.7 ± 4.6	225.5 ± 12
Dermal					
Broilers	44.5 ± 3.0	40.7 ± 2.6	41.7 ± 2.0	53.5 ± 2.8	58.1 ± 4.0
RF	55.7 ± 4.5 *	110.9 ± 17 *	131.9 ± 3.5 *	90.5 ± 3.7 *	69.4 ± 16.1
Hypodermal					
Broilers	1854 ± 167	2191 ± 160	3026 ± 158	1127 ± 56	2101 ± 127
RF	2023 ± 189	2366 ± 192	2057 ± 134 *	1040 ± 28 *	1526 ± 171 *

After bloodletting, collected skin samples before scalding were fixed in formalin and then used for stratum corneum, epidermal, dermal, and hypodermal layer thickness measurement under microscopic observations. Results are expressed as means ± SD (*n* = 10). * significant difference vs. broilers within the same skin part and skin layer (*p* < 0.05). RF, Red-Feather country chickens.

**Table 4:** Skin collagen content in different parts of the body in broilers and RF chickens.

Staining Intensity	Scalp	Neck	Back	Chest	Abdomen
Broilers	21.2 ± 4.8	12.0 ± 1.6	18.7 ± 4.6	25.4 ± 8.1	7.5 ± 0.7
RF	22.8 ± 6.6	16.1 ± 1.7 *	20.3 ± 6.3	24.0 ± 5.4	25.2 ± 1.0 *

After bloodletting, collected skin samples before scalding process were fixed in formalin and then used for Masson’s trichrome staining for collagen quantification using Image-J software. Results are expressed as means ± SD (*n* = 10). * significant difference vs. broilers within the same column (*p* < 0.05). RF, Red-Feather country chickens.

**Table 5:** Wing and tail feather calamus depth and diameter in broilers and RF chickens.

Unit (cm)	1st SWF	5th SWF	1st MTF	5th MTF
Depth				
Broilers	2.40 ± 0.32	2.51 ± 0.31	2.34 ± 0.46	2.00 ± 0.37
RF	2.41 ± 0.23	2.55 ± 0.39	1.31 ± 0.30 *	1.84 ± 0.16
Diameter				
Broilers	0.28 ± 0.016	0.21 ± 0.016	0.23 ± 0.034	0.22 ± 0.026
RF	0.25 ± 0.048	0.31 ± 0.032 *	0.19 ± 0.026	0.23 ± 0.021

After bloodletting, the 1st and 5th secondary wing feather of both right and left side and those of the main tail feathers (not the sickle feathers) were used for feather calamus diameter and depth measurement. Results are expressed as means ± SD (*n* = 10). * significant difference vs. broilers within the same column and measurement (*p* < 0.05). RF; Red-Feather country chickens. SWF, secondary wing feathers; MTF, main tail feathers.

**Table 6:** Effects of scalding on the denaturation of hip skins in broilers and RF chickens.

	Soft Scalding (57 °C/120 s)						Hard Scaling (60 °C/60 s)						SEM		
	Broilers			RF			Broilers			RF					
Degree of denaturation															
(distance of color change, μm)	125			107 *			199 ^#^			149 * ^#^			3.1		
	*p*-value														
Source of variation	Main Effect								Interaction						
	Scalding				Breed				Scalding × Breed						
	<0.001				0.013				0.21						

After scalding, hip skins were collected and fixed in formalin for H&E staining. The distance of color changes from the outer epidermal (dark purple) to the inner dermal layer (bright pink purple) was measured under microscopic observations for the degree of denaturation by scalding. The two-way analysis of variance (ANOVA) using scalding (hard vs. soft) × breed (broilers vs. RF chickens) was performed to analyze the main effects and their interaction. * significant difference vs. broilers within the same scalding method (*p* < 0.05). ^#^ significant difference vs. soft scalding within the breed (*p* < 0.05). RF, Red-Feather country chickens.

**Table 7:** Effects of scalding on the gap distance between the feather and follicle sheath in the wing and tail feather in broilers and RF chickens.

	Before Scalding		After Soft Scalding		After Hard Scalding	
Unit (μm)	Broiler	RF	Broiler	RF	Broiler	RF
3rd SWF	2.4 ± 3.5 ^c^	≌0 ^z^	17.9 ± 6.9 ^b^	15.4 ± 1.8 ^y^	50.4 ± 8.8 ^a^	32.0 ± 6.5 * ^x^
7th SWF	8.5 ± 1.2 ^c^	1.5 ± 2.1 * ^z^	28.1 ± 7.3 ^b^	19.0 ± 1.1 * ^y^	54.7 ± 10.1 ^a^	27.7 ± 7.5 * ^x^
3rd MTF	5.2 ± 3.2 ^c^	≌0 * ^z^	30 ± 15.5 ^b^	18.9 ± 2.1 * ^y^	83.2 ± 11.1 ^a^	23.6 ± 5.3 * ^x^
7th MTF	1.2 ± 0.8 ^c^	0.67 ± 1.0 ^z^	23.7 ± 7.0 ^b^	15.8 ± 0.5 * ^y^	64.9 ± 12.1 ^a^	21.0 ± 0.6 * ^x^

Immediately after bloodletting, or undergone soft or hard scalding, the 3rd and 7th feather of both right and left secondary wing feathers and those of the main tail feathers (not the sickle feathers) containing the surrounding cutaneous tissues were collected for fixation and H & E staining. The gap distances between the surface of feather and follicle sheath were measured under microscopic observations. Results are expressed as means ± SD (*n* = 10). * significant difference vs. broilers within the same row and scalding method (*p* < 0.05). Means with different superscripts within the same row and breed differ significantly (^a–c^ for broilers, ^x–z^ for RF chickens, *p* < 0.05). RF, Red-Feather country chickens.

## Data Availability

The data presented in this study are available on request from the corresponding authors.

## References

[1] Fletcher D.L., Thomason D.M. (1980). The influence of environment and processing conditions on the physical carcass quality factors associated with oily bird syndrome. Poult. Sci..

[2] Jones J.M., Grey T.C., Mead G.C. (1989). Influence of processing on product quality and yield. Processing of Poultry.

[3] Buhr R.J., Walker J.M., Bourassa D.V., Caudill A.B., Kiepper B.H., Zhuang H. (2014). Impact of broiler processing scalding and chilling profiles on carcass and breast meat yield. Poult. Sci..

[4] Jeong J.Y., Janardhanan K.K., Booren A.M., Karcher D.M., Kang I. (2011). Moisture content, processing yield, and surface color of broiler carcasses chilled by water, air, or evaporative air. Poult. Sci..

[5] McKee S.R., Townsend J.C., Bilgili S.F. (2008). Use of a scald additive to reduce levels of Salmonella Typhimurium during poultry processing. Poult. Sci..

[6] Dikeman M., Devine C., Schilling M.W., Vizzier-Thaxton Y., Alvarado C.Z. (2014). Slaughter-Line of Operation|Poultry. Encyclopedia of Meat Sciences.

[7] Sams A.R., McKee S.R., Owens C.M., Alvarado C.Z., Sams A.R. (2010). First processing: Slaughter through chilling. Poultry Meat Processing.

[8] Bowker B.C., Zhuang H., Buhr R.J. (2014). Impact of carcass scalding and chilling on muscle proteins and meat quality of broiler breast fillets. LWT Food Sci. Technol..

[9] Harris C.E., Gottilla K.A., Bourassa D.V., Bartenfeld L.N., Kiepper B.H., Buhr R.J. (2018). Impact of scalding duration and scalding water temperature on broiler processing wastewater loadings. J. Appl. Poult. Res..

[10] Chao C.H., Huang Y.M., Chen C.F., Ho Y.C., Su M.L., Lee Y.P. (2005). The growth performance of commercial red-feathered and black-feathered Taiwan country chicken. J. Chin. Soc. Anim. Sci..

[11] Shung C.C., Hsin K.Y., Tan F.J., Chen S.E. (2022). Effects of hard and soft scalding on defeathering and carcass quality of different breeds of chickens. Animals..

[12] Leeson S., Walsh T. (2004). Feathering in commercial poultry I. Feather growth and composition. World’s Poult. Sci. J..

[13] Yu M., Wu P., Widelitz R.B., Chuong C.M. (2002). The morphogenesis of feathers. Nature.

[14] Yu M., Yue Z., Wu P., Wu D.Y., Mayer J.A., Medina M., Widelitz R.B., Jiang T.X., Chuong C.M. (2004). The developmental biology of feather follicles. Int. J. Dev. Biol..

[15] Lin S.J., Wideliz R.B., Yue Z., Li A., Wu X., Jiang T.X., Wu P., Chuong C.M. (2013). Feather regeneration as a model for organogenesis. Dev. Growth Differ..

[16] Waller J.M., Maibach H.I. (2006). Age and skin structure and function, a quantitative approach (II): Protein, glycosaminoglycan, water, and lipid content and structure. Skin Res. Technol..

[17] Turunen M.J., Khayyeri H., Guizar-Sicairos M., Isaksson H. (2017). Effects of tissue fixation and dehydration on tendon collagen nanostructure. J. Struct. Biol..

[18] Onul A., Colvard M.D., Paradise W.A., Elseth K.M., Vesper B.J., Gouvas E., Deliu Z., Garcia K.D., Pestle W.J., Radosevich J.A. (2012). Application of immunohistochemical staining to detect antigen destruction as a measure of tissue damage. J. Histochem. Cytochem..

[19] Ricard-Blum S. (2011). The collagen family. Cold Spring Harb. Perspect Biol..

[20] Berg R.A., Prockop D.J. (1973). The thermal transition of a non-hydroxylated from of collagen, evidence for a role for hydroxyproline in stabilizing the triple-helix of collagen. Biochem. Biophys. Res. Commun..

[21] Xu F., Lu T.J., Seffen K.A. (2009). Thermally-induced change in the relaxation behavior of skin tissue. J. Biomech. Eng..

[22] Arnoczky S.P., Aksan A. (2000). Thermal modification of connective tissues: Basic science considerations and clinical implications. J. Am. Acad. Orthop. Surg..

[23] Ong B.B., Payne-James J. (2005). Injury, fatal and nonfatal|Burns and scalds. Encyclopedia of Forensic and Legal Medicine.

[24] Kafri I., Cherry J.A., Jones D.E., Siegel P.B. (1985). Breaking strength and composition of the skin of broiler chicks: Response to dietary calorie-protein ratios. Poult Sci..

[25] Weinberg Z.G., Angel S., Navrot C. (1986). The effects of sex, age, and feed on tensile strength of broiler skin. Poult. Sci..

[26] Perez-Puyana V., Ostos F.J., López-Cornejo P., Romero A., Guerrero A. (2019). Assessment of the denaturation of collagen protein concentrates using different techniques. Biol. Chem..

[27] Silver F.H., Freeman J.W., DeVore D. (2001). Viscoelastic properties of human skin and processed dermis. Skin Res. Technol..

[28] Suurs P., van den Brand H., Farawu K., Daamen W.F., Barbut S. (2023). Effects of broiler weight and strain on skin collagen characteristics and their applicability for co-extruded sausage casings. Food Struct..

[29] Burjanadze T.V. (1979). Hydroxyproline content and location in relation to collagen thermal stability. Pept. Sci..

[30] Arda O., Göksügür N., Tüzün Y. (2014). Basic histological structure and functions of facial skin. Clin. Dermatol..

[31] Mallet C., Souci L., Ledevin M., Georgeault S., Larcher T., Denesvre C. (2022). Establishment of a culture model for the prolonged maintenance of chicken feather follicles structure in vitro. PLoS ONE.

[32] Mattiello S., Guzzini A., Del Giudice A., Santulli C., Antonini M., Lupidi G., Gunnella R. (2022). Physico-chemical characterization of keratin from wool and chicken feathers extracted using refined chemical methods. Polymers.

[33] Wang B., Yang W., McKittrick J., Meyers M.A. (2016). Keratin: Structure, mechanical properties, occurrence in biological organisms, and efforts at bioinspiration. Prog. Mater. Sci..

[34] Alibardi L. (2007). Keratinization of sheath and calamus cells in developing and regenerating feathers. Ann Anat..

[35] Yao D., Zhang D., He M., Yin G., Cui Y. (2016). The structure, tensile properties and water resistance of hydrolyzed feather keratin-based bioplastics. Chin. J. Chem. Eng..

